# Subcellular Phenotyping: Using Proteomics to Quantitatively Link Subcellular Leaf Protein and Organelle Distribution Analyses of *Pisum sativum* Cultivars

**DOI:** 10.3389/fpls.2019.00638

**Published:** 2019-05-17

**Authors:** Sebastian Schneider, Dominik Harant, Gert Bachmann, Thomas Nägele, Ingeborg Lang, Stefanie Wienkoop

**Affiliations:** ^1^Division of Molecular Systems Biology, Department of Ecogenomics and Systems Biology, University of Vienna, Vienna, Austria; ^2^Core Facility Cell Imaging and Ultrastructure Research, University of Vienna, Vienna, Austria; ^3^Department Biology I, Plant Evolutionary Cell Biology, Ludwig-Maximilians Universität, Munich, Germany

**Keywords:** organelle marker peptides, organelle stoichiometry, field pea, Mass Western, 3D Confocal Laser Scanning Microscopy

## Abstract

Plant phenotyping to date typically comprises morphological and physiological profiling in a high-throughput manner. A powerful method that allows for subcellular characterization of organelle stoichiometric/functional characteristics is still missing. Organelle abundance and crosstalk in cell dynamics and signaling plays an important role for understanding crop growth and stress adaptations. However, microscopy cannot be considered a high-throughput technology. The aim of the present study was to develop an approach that enables the estimation of organelle functional stoichiometry and to determine differential subcellular dynamics within and across cultivars in a high-throughput manner. A combination of subcellular non-aqueous fractionation and liquid chromatography mass spectrometry was applied to assign membrane-marker proteins to cell compartmental abundances and functions of *Pisum sativum* leaves. Based on specific subcellular affiliation, proteotypic marker peptides of the chloroplast, mitochondria and vacuole membranes were selected and synthesized as heavy isotope labeled standards. The rapid and unbiased Mass Western approach for accurate stoichiometry and targeted absolute protein quantification allowed for a proportional organelle abundances measure linked to their functional properties. A 3D Confocal Laser Scanning Microscopy approach was developed to evaluate the Mass Western. Two *P. sativum* cultivars of varying morphology and physiology were compared. The Mass Western assay enabled a cultivar specific discrimination of the chloroplast to mitochondria to vacuole relations.

## Introduction

Modern plant phenotyping is quantitatively linking molecular and biochemical data with the plants morphological, physiological and agronomic parameters. In general, plant phenotyping aims to improve productivity and stress tolerance. One major challenge is the ability to screen large populations in a high-throughput manner. However, to date microscopy cannot be considered high-throughput, making fast screening of several biological replicates and plant accessions impossible. Hence, there is noticeable lack in organelle size and distribution analyses.

Cells of living organisms are surrounded by a membrane and contain various sub-structures and organelles. Each of these organelles has a characteristic morphology and processes specific function(s). In general, organelles such as mitochondria, grow with the cell to accommodate an increased need for their functions (Marshall, 2012). Others may also increase in number such as described for peroxisomes (Chan and Marshall, 2010; Marshall, 2012). Furthermore, cell organelles and structures relate to each other depending on cell requirements. Their relations depend on their cellular positions, surface areas and volumes through varying size, shape, or number. Upon developmental or environmental changes cells and organelles process adaptation. This adaptation includes the re-distribution of metabolites as a crosstalk between the organelles and molecular plasticity of specific organelle functions. One of the best studied examples in plants is the interconnection existing between mitochondria, chloroplasts and peroxisomes (Schnarrenberger and Fock, 1976). They coordinate their activities as a function of photorespiration during drought stress.

Mitochondria, chloroplasts and vacuoles occupy the largest portion of photosynthetically active mesophyll cells of plant. While their major functions are well described, not much attention has been paid to their cellular volumes and the role of their relative variation in abundance between plant species and environmental adaptation processes. Using light and electron microscopy, the subcellular volumes of these organelles were roughly determined for barley and spinach leaves (Winter et al., 1993, 1994) as well as potato (Leidreiter et al., 1995). According to those studies, volume differences for chloroplasts to mitochondria range between 10- and 20-fold and vacuole to chloroplasts around 10-fold. Miroslavov and Kravkina (1991) used microscopy to describe a change in chloroplast and mitochondria number of various plant species naturally occurring in mountainous regions at increasing levels of altitude. Independent of the plant species they examined, the number of mitochondria increased with altitude. In a more recent study, volumes of chloroplast fine structures, mitochondria, and peroxisomes from control and drought-stressed spinach leaves were analyzed by transmission electron microscopy (Zellnig et al., 2004). They found that in stressed plants, mitochondria had only 65% of the volume compared to controls. Furthermore, Armstrong et al. (2006) investigated changes in mitochondrial abundance by linking ultrastructure and respiration activity measurements in Arabidopsis and found a heterogeneity of mitochondrial structure and abundance within the leaf tissue. These few studies underline that organelle plasticity is actively involved in plants acclimation processes. However, an integrative approach linking functional organelle data with molecular processes is still missing.

Proteomics can provide insight into the specialized biochemistry of distinct organelles and has been widely used to investigate and evaluate subcellular localizations of e.g., mitochondria (Huang et al., 2014), chloroplasts (Ferro et al., 2010), and vacuole (Carter et al., 2004). Mitochondria and chloroplasts are surrounded by a double membrane, while vacuoles are bordered by a single membrane system. Many of their membrane specific proteins such as ATPases, with key organelle functions, have been identified and have successfully been applied as markers for organelle purification, function and subcellular localization studies (Lang et al., 2011; LaMontagne et al., 2016). For the determination of organelle specific protein distributions, a non-aqueous fractionation (NAF) technique has previously been applied (Arrivault et al., 2014). This approach was initially used for the analysis of metabolite allocation studies across organelles (Gerhardt and Heldt, 1984) but also enables an in depth integrative investigation of subcellular metabolomics and proteomics. NAF has been described to be especially powerful to discriminate chloroplast, vacuolar, and cytosolic compounds (Fürtauer et al., 2016).

Functional and subcellular annotation of plant proteins enables targeted relative organelle abundance profiling (Parsons and Heazlewood, 2015) now called multiple marker abundance profiling (MMAP) (Hooper et al., 2017). This strategy integrates data dependent mass spectrometry acquisition with selective reaction monitoring (SRM). It allows for a rapid, relative estimation of organelle abundance changes. A challenge, which remains is the estimation of organelle abundance relative to each other and among different populations. The SRM approach is used to specifically target proteotypic peptides and is restricted to accurate relative quantification. The introduction of stable isotope labeled peptides (Barr et al., 1996) in combination with SRM is called Mass Western (Lehmann et al., 2008). It enables absolute quantification and thus a stoichiometric analysis of different proteins, isoforms and subunits andhas recently been improved by the integration of a concatenated synthetic peptide system (Recuenco-Muñoz et al., 2015).

In order to approach the relationship between organelle abundances and functions, we developed a Mass Western kit for organelle stoichiometry investigations of *Pisum sativum* leaves. We used the NAF technique to define specific organelle membrane marker peptides of *P. sativum* (OMMPOPs) for vacuole, mitochondria and chloroplast. The concatenated, stable isotope marker peptide assay was employed to rapidly estimate the relative organelle stoichiometry of two pea cultivars, different in their growth performance, which is expressed by the abundance of organelle-membrane specific and functionally crucial proteins.

## Materials and Methods

### Plant Growth Conditions

*Pisum sativum* plants (cv. Messire and Protecta) were cultivated under glasshouse conditions at a temperature of 15°C and a relative humidity of 60% (±5%) in a 14 h light and 10 h dark photoperiod as described previously (Desalegn et al., 2016; Turetschek et al., 2017). The average Photosynthetically Active Radiation (PAR) across the plant and day cycle was around 150 μmoles m^2−1^ s^−1^ (μE) (max. ~600 μE). Altogether, there were 3 plants per pot and 10 pots per plant and cv. Plant leaves were harvested and studied 4 weeks after germination. The length of two internodes of five plants per cultivar were measured for the evaluation of the most characteristic morphological difference. For protein absolute quantification, two analyses were carried out. In the first analysis, all leaves across the plants were analyzed, while in a second analysis only the youngest, fully developed leaves were investigated.

### Physiological Measurements

Chlorophyll content was measured using a Minolta SPAD®, photosynthesis based parameters (quantum yield, non-photochemical quenching) as well as transpiration cooling and water content of the leaves were monitored using a PhotosynQ® MultispeQ v1.

### Fluorescent Markers and Labeling

The mitochondria of leaf cross sections of *Pisum sativum* were stained using the fluorescence dye MitoTracker (Invitrogen; working concentration: 1 μM, incubation time: 45–60 min). Additionally, the cytoplasm was labeled with fluorescein (Sigma Aldrich; 5 mg/l, incubation time 10–30 min). leading to a good visualization of the vacuolar cavity for 3D reconstructions and quantification. The dyes were added directly to the cross sections on a coverslip. During incubation, the samples were stored at room temperature in a petri dish with a moist filter paper, covered with aluminum foil to prevent bleaching. For chloroplasts, auto-fluorescence was used. The mitochondrial fluorescence signal in combination with the auto-fluorescence of the chloroplasts of the mesophyll cells were utilized for organelle area and volume determination.

### Confocal Laser Scanning Microscopy (CLSM)

All images were taken at a confocal laser scanning microscope (Leica DMRE SP5) with an argon laser for excitation of fluorescence signals (MitoTracker: ex/em 488/520–550 nm; chloroplasts: ex/em 514/570–600 nm; fluorescein: ex/em 488/520–530 nm). Additionally, bright field images were taken in transmission mode. The scanning speed was 400 Hz and we used a 63x (NA 1.2) water immersion objective for all images. The focal depth was about 0.5 μm and the pinhole was set to one Airy disc. Stacks of 10 to 36 optical sections per series were recorded to cover a total cell layer and the fluorescence data jointly used as maximum projections. For 3D reconstructions, up to 180 sections were recorded according to the voxel size of the z-stack. Volume rendering and 3D visualizations (videos) were performed at an AMIRA® workstation (v6.2.0, FEI).

### Quantification of Confocal Fluorescence Intensity

GSA-Image Analyzer v4.0.9 (GSA GmbH) was used to calculate the organelle areas. Fluorescence limits and object area recognition were utilized to determine the respective chloroplast and mitochondrion parts. Leaf cross sections of two different genotypes (3 biological replicates of Messire and Protecta) were analyzed. To get a complete picture of the entire cellular components, successive optical sections were taken and merged into 2D stack as maximum projection using Adobe Photoshop. Additionally, the cellular outlines were utilized to cut out regions for the fluorescence signal quantification, especially to avoid the inclusion of background chlorophyll fluorescence signals, in the leaf cross sections.

### Overview of the Non-aqueous Fractionation (NAF) Experimental Design

**Figure 1:**
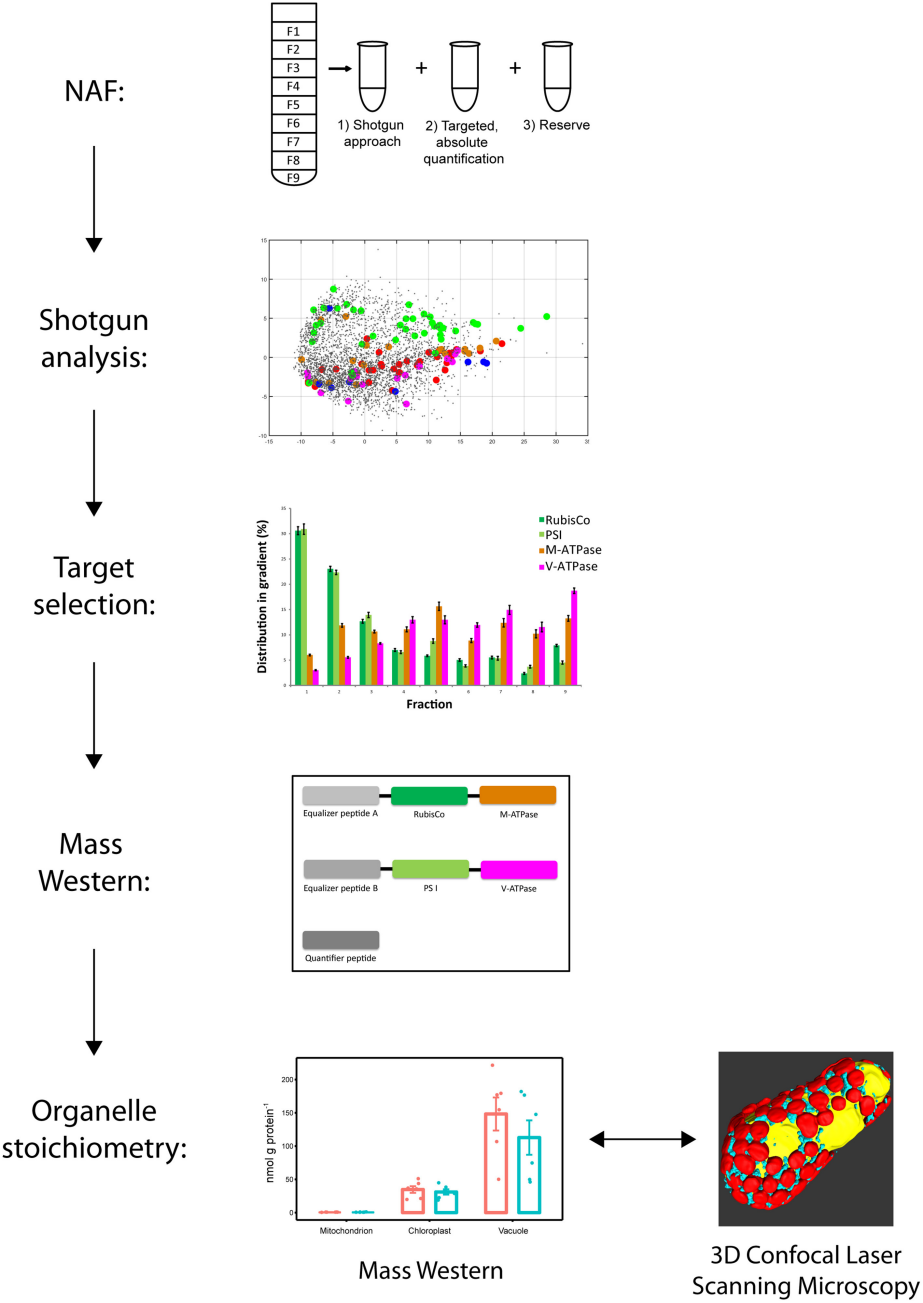
Workflow for establishment of the organelle stoichiometry assay. Non-aqueous fractionation (NAF) was used to fractionate leave organelles of *Pisum sativum* cultivars. LC-MS/MS shotgun analyses of the NAF fractions revealed organelle membrane marker proteins of mitochondria, chloroplasts and vacuole. Proteotypic peptide sequences were selected from the organelle marker proteins and synthesized to get concatenated isotopic labeled peptide standards (Organelle Membrane Marker Peptides of *P. sativum* = OMMPoPs). A Mass Western was performed using the OMMPoPs, spiked into a crude protein leaf extract for absolute quantification and determination of relative organelle stoichiometry. For comparison, relative organelle stoichiometry of mitochondria and plastids were also calculated from microscopy.

The experiment was structured in several steps (Figure 1). In the first step, leaf suspensions of 2 week old plants including proteins were subcellular fractionated using non-aqueous fractionation (NAF). Then proteins where extracted and fractions analyzed by shotgun liquid chromatography mass spectrometry (LC-MS/MS). Identified proteins were verified for organelle targeting and proteotypic characterization. A Mass Western approach was set up. Specificity of mitochondrial marker was re-evaluated. A Mass Western analysis for organelle stoichiometric analysis was conducted including a clod stress experiment and a cross-check of organelle abundances was carried out using confocal microscopy and 3D reconstruction.

### Non-aqueous Fractionation (NAF)

Altogether, NAF gradients of 20 biological replicates were prepared (10 plants of cultivar Messire and 10 of cultivar Protecta). The non-aqueous fractionation was performed according to Nägele and Heyer (2013): The freeze-dried plant material was resuspended in heptane-tetrachloroethylene and sonified on ice. The suspension was filtered, centrifuged and the pellet was resuspended. In a non-aqueous density gradient of heptane and tetrachloroethylene, the suspension was fractionated by ultracentrifugation. Nine fractions of 1 mLwere collected, each divided into subfractions of 0.3 mL protein extraction.

### Protein Extraction

The nine different fractions of the gradient were each resuspended in Urea buffer (50 mM HEPES, 8 M Urea, pH 7.8) and afterwards centrifuged (10,000 × g, 10 min, 4°C). The supernatants were transferred into new microfuge tubes in ice-cold aceton with 0.5% β-mercaptoethanol. The tubes were vortexed and stored over night at −20°C to precipitate the proteins. Finally, 8 M Urea buffer was used to resuspend the pellet for digestion.

### Protein Digestion and Desalting

Protein concentration was determined via Bradford assay (Bradford, 1976) using BSA as a standard. The extracted proteins (50 μg) were digested using LysC (1:100 v/v, 5 h, 30°C, Roche, Mannheim, Germany). Afterwards the buffer was diluted to 2 M Urea concentration with trypsin buffer [10 % (v/v) ACN, 100 mM AmBic, 1 mM CaCl2, 5 mM DTT] incubated over night at 37°C with Poroszyme immobilized trypsin beads (1:10, v/v; Applied Biosystems, Darmstadt, Germany).

Desalting was performed with Pierce C18 stage tips (Thermo Fisher Scientific, 100 μL bed volume) according to Ishihama et al. (2006). The digested protein solutions were centrifuged for 2–3 min at 5,000 × g at 4°C to spin down the trypsin beads and then formic acid (FA) was added to a final concentration of 3% (v/v) FA in the solution. The C18 resin of the stage tips was first equilibrated with 100 μl methanol and afterwards washed twice with 100 μl 0.1 % (v/v) FA. After that the samples were pipetted through the stage tips to ensure that all protein bound to the C18 material. Subsequently samples were desalted by washing twice with 100 μl of 0.1% (v/v) FA. Finally, proteins were eluted into low-bind microtubes two times with 100 μl MeOH. For safe storage, the samples were dried down and stored at −20°C.

### Nano ESI LC-MS/MS Analysis of NAFs

The fractioned proteins were dissolved in 2% (v/v) ACN, 0.1% (v/v) FArandomized and 0.5 μg of each sample were loaded on a reverse phase C18 column (PepMap®RSLC, Thermo scientific, 2 μm particle size) and separated during a 90 min gradient with a flow rate of 300 nL min^−1^ (Ulti-Mate 3000, Thermo Fisher Scientific, Austria). MS measurement was performed on an LTQ-Orbitrap Elite (Thermo Fisher Scientific, Bremen, Germany) with the following settings: Full scan range 350–1,800 m/z, max. 20 MS2 scans (activation type CID), repeat count 1, repeat duration 30 s, exclusion list size 500, exclusion duration 60 s, charge state screening enabled with rejection of unassigned and +1 charge states, minimum signal threshold 1,000.

### Protein Identification and Label Free Quantification

Thermo raw files were used to identified and relatively quantified proteins in MaxQuant (Cox and Mann, 2008) version 1.6.0.1. An in-house database of *Pisum sativum* and standard identification parameters were used as described previously (Turetschek et al., 2016). In short: first search peptide tolerance 20 ppm, main search tolerance 4.5 ppm/6 ppm, ITMS MS/MS match tolerance 0.8 Da, intensity threshold 5,000. All files were searched with the following variable modifications: oxidation of methionine and acetylation of the N-term. Maximum two missed cleavages were allowed. A retention time window of 20 min was used to search for the best alignment function and identifications were matched between runs in a window of 0.7 min. A revert decoy db was used to set a cut-off at a FDR of 0.01 (at PSM and protein level). A minimum of 6 amino acids was required for identification of a peptide and at least two different peptides necessary for protein identification. Label free quantification (LFQ) was done when at least one MS2 scan was present. LFQ minimum ratio was set to 2. Stabilization of large LFQ ratios was active.

### Heavy Isotope Labeled Organelle-Marker Peptides for Mass Western

After NAF analyses, organelle affiliation of proteins was based on functional annotations and cross-evaluated using TargetP (Emanuelsson et al., 2000) and WoLF PSORT protein localization predictor (Horton et al., 2007) after NAF analyses. Subsequently, marker-peptides were selected according torobustness and abundance of MS signals and mutual exclusion quantification (Picotti and Aebersold, 2012; Lyon et al., 2014). We previously described the selective peptide extraction strategy (SELPEX) (Castillejo et al., 2014). Here, our targets have been reproducibly identified across all replicates of each cultivar. In addition, these “organelle membrane -marker peptides of *Pisum sativum*” (OMMPoPs) are proteotypic peptides of organelle specific, membrane-integral proteins that play a major functional role for each organelle. For functional organelle analysis, additionally, a marker peptide for the large subunit of RuBisCO was used. Details about peptide sequences used for the Mass Western can be found in the Table S1.

As previous described, selected peptides were labeled with one heavy amino acid each (heavy isotopes: 13C/15N) while the two equalizer peptides (EP) were double labeled at different positions (Table S1) to distinguished them from the quantifier peptide (QP) (Recuenco-Muñoz et al., 2015). The (QP) was added as a third internal standard in order to achieve an optimal absolute quantification. This QP was a mono-labeled peptide with the same sequence as the equalizer peptides, which was synthesized as “AQUA ULTIMATE”–peptide (precision equal or better ±5%, Thermo Scientific Heavy Peptide AQUA Custom Synthesis Service, Ulm, Germany) and can be stoichiometrically quantified with very high accuracy (Recuenco-Muñoz et al., 2015).

### Mitochondria Enrichment for Marker Peptide Evaluation

Mitochondria isolation, including homogenization, filtering, and differential centrifugation as well as density gradient centrifugation was carried out according to Huang et al. (2014). About 30 g *Pisum sativum* leaf material was used from cultivar Protecta. For evaluation of the selected mitochondrial marker peptide, Mass Western was applied to the extracted mitochondrial fraction and compared to the vacuolar marker peptide.

### Absolute Quantification and Organelle Protein Stoichiometry Calculations

Protein extraction, digestion and desalting of whole Pisum leaves was performed as described for the NAF fractions but with addition of synthetic standard peptides: For absolute quantification the synthetic standard peptides were digested and spiked (Ahsan et al., 2018) at known concentrations (100 fmol per μg) into each sample and measured on a one-dimensional (1D) nano-flow LC system (Ulti-Mate 3000, Thermo Fisher Scientific), coupled to an Orbitrap Elite mass spectrometer (Thermo Scientific). Peptides were eluted using an Easy-Spray RSLC PEPMAP® C18 column (15 cm × 50 μm, 2 μm; Thermo Scientific) during a 30 min gradient from 2 to 50% (v/v) acetonitrile containing 0.1% (v/v) FA with a controlled flow rate of 0.3 μL min^−1^. MS analysis was performed in the positive profile mode within mass range from 450 to 800, the range of the doubly charged target peptide masses. Additionally, a calibration curve of all standard peptides was prepared ranging from 1 to 500 fmol (1, 10, 50, 100, 250, 500) (Lyon et al., 2014; Recuenco-Muñoz et al., 2015). Due to an unambiguous identification based on identical retention times and the accurate m/z-values of the labeled and native peptides, base peak areas of the extracted precursor ions were used for quantification. The extracted protein amounts determined by the Bradford-assay were used to calculate the absolute amount of protein per sample.

Organelle abundance and stoichiometry analysis was calculated by peak integration (Xcalibur) of the respective native (non-labeled) to standard (labeled) areas. Since the synthetic standard peptides are in a stoichiometric dependence (1:1:1), which does not represent the stoichiometry of the native sample, a standard curve was prepared and peptide peak areas to abundance checked for linearity. Where necessary, standard peak areas of respective abundances to the native areas were compared to the standard curve. As the ratio of the two equalizer peptides (EP) was found to be 1:1 (±10%) additional balancing was not necessary. Moreover, mitochondrial ATPase and the chloroplast photosystem I marker enzymes are inner membrane proteins. Assuming that these enzyme abundances are closely proportional to membrane/organelle abundance (volume), we further normalized these enzyme abundances with a factor, reflecting the proportion of inner to outer membrane systems of the different organelles, which was previously published to be about 3-fold for the mitochondria and about 11-fold for the chloroplasts (Schwerzmann et al., 1986; Zellnig et al., 2004; John et al., 2005).

### Statistical Analysis

PCA (principal component analysis) and ANOVA (analysis of variance) of NAF data were carried out using the MatLab tool COVAIN (Sun and Weckwerth, 2012). For statistical analyses of morphologic, microscopic and proteomic data, a Student *t*-test was performed with Excel Microsoft Office, 2010. Excel was also used to create diagrams, and calculated standard deviations and *p*-values of the student *t*-test. For physiological measurements, results were given as bar graphs featuring individual confidence intervals. Kruskal-Wallis error probabilities were calculated using Statgraphics Centurion 18® (Statgraphics Technologies Inc.).

## Results

### Physiological and Morphological Phenotype

Differences in the morphology between the two cultivars are show in Figure S1. Protecta is growing faster as demonstrated in the picture and reflected by the internode length (Figure S1). Also the leaf protein content per FW was significantly higher in Protecta compared to Messire (Figure S1). Physiological parameters revealed that the Chlorophyll content of the cultivars did not differ significantly (Figure S2). While Fv/Fm was slightly significantly (*p* < 0.05) higher (5–10%) in Messire., Protecta invested 10% more energy in repair processes (NPQt, non-photochemical quenching). Messire lost ~3% more energy to thermal dissipation (PhNO). Water content (absorbance at 940 nm) and cooling of the leaf surface by evapotranspiration (leave temperature difference) was significantly higher (*p* < 0.005) in Messire leaves, with about 5 and 30%, respectively (Figure S2).

### Organelle Quantification by 3D Reconstruction

**Figure 2:**
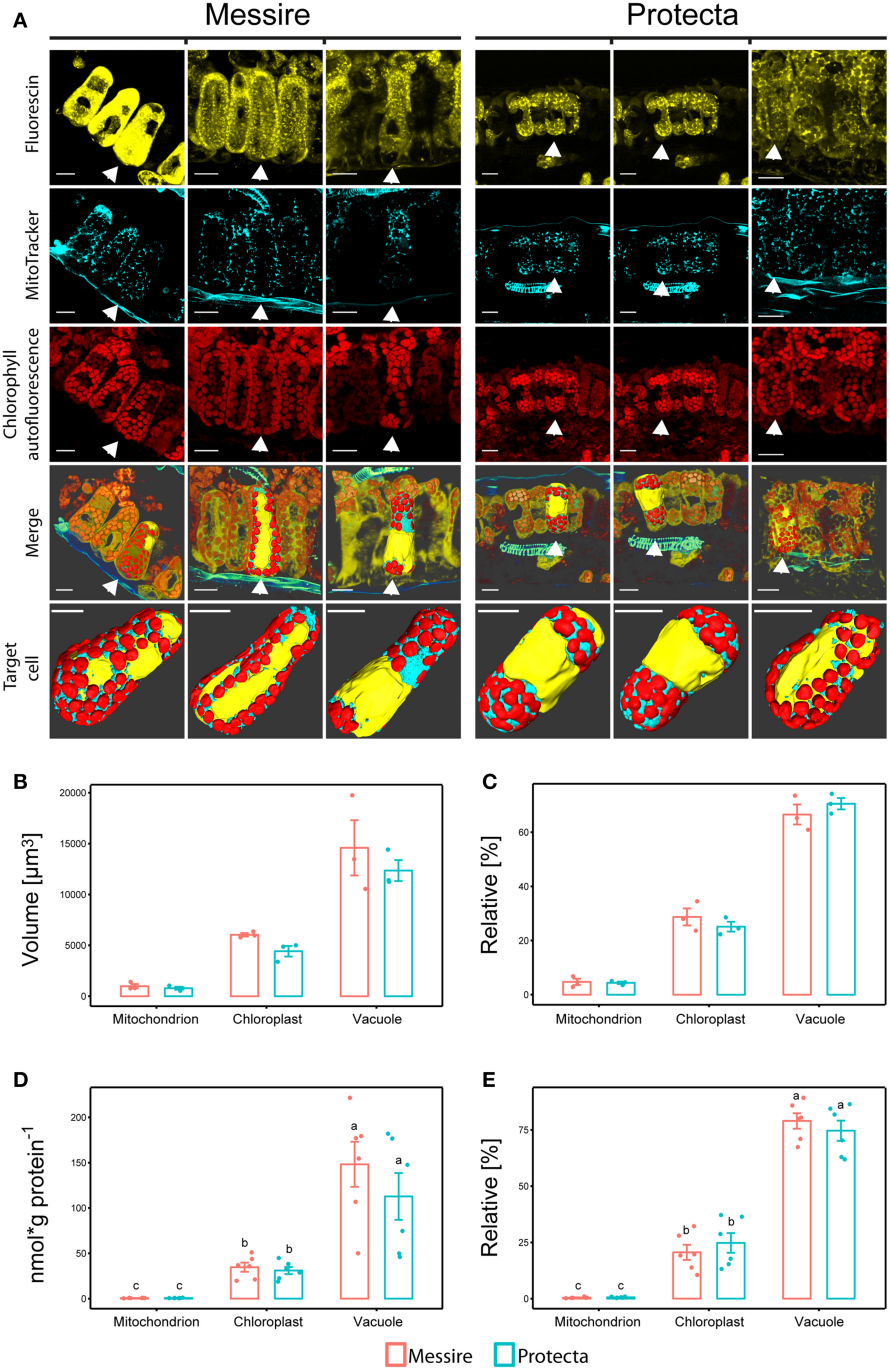
**(A)** Confocal microscopy and 3D reconstructions of palisade layer of *P. sativa* cultivars. Messire and Protecta. 1st row: cytoplasm labeled with fluorescein, 2nd row: mitochondria labeled with MitoTracker orange®, 3rd row: autofluorescence of chlorophyll. 4th row: merge of three channels and surface rendering of one selected cell; 5th row: isolated cells (with vaculole cavity labeled in yellow) of rows 1–4: 3D reconstruction and surface rendering. Unlabeled bars: 20 μm. 3D videos in supplementals. **(B,C)** Comparison of organelle abundances of pea cultivars using confocal microscopy. **(B)** Volume [μm^3^] and **(C)** relative volume [%], *n* = 3; Significant differences at *p* < 0.05 are indicated by different letters (Kruskal Wallis); error bars = standard deviation. **(D,E)** Mass Western: comparison of absolute and relative organelle-protein marker and abundances of pea cultivars (total leaf extract). **(D)** Absolute protein abundances expressed as [nmol (g protein per g FW)^−1^] and [nmol g protein^−1^], **(E)** relative marker protein abundances [%]; Significant differences at *p* < 0.05 are indicated by different letters, *n* = 5 (Kruskal Wallis); error bars = standard deviation.

Using confocal laser scanning microscopy (CLSM) and specific organelle fluorescence markers, a computational reconstruction of organelle abundances (area and volume) within leaf cells of the palisade parenchyma was possible (Figure 2A; Table S2 and Videos 1–6). The ratio between chloroplast and mitochondria was on average about 8-fold and between vacuole and chloroplast about 2.5-fold for both pea cultivars. The relative distribution (sum of all organelle volumes set to 100%) of chloroplast: vacuole: mitochondria for Protecta was about 25%: 70%: 5% and for Messire about 28%: 67%: 5% (Figure 2C). As a result, this data showed no significant difference between the cultivars.

Altogether, volumes (μm^3^) of all measured organelles of cultivar Messire were larger (on average about 1.2-fold) then of Protecta, however, only volumes of chloroplasts were significantly different (*p* < 0.05) between Protecta and Messire (1.4-fold) (Figure 2B). Area values (μm^2^) did not show any statistically significant difference between the cultivars (Table S2).

### Organelle-Protein Fractionation and High-Confidence Subcellular Peptide Marker Lists Selection

The non-aqueous fractionation (NAF) approach was carried out with 10 biological leaf samples. Proteins of 9 fraction from 10 different NAF gradients were extracted, digested and measured using LC-MS/MS. In order to obtain an overview of the subcellular distribution of the leaf proteome and NAF performance, the relative peptide MS intensities were analyzed. For this purpose proteins, containing proteotypic peptides where selected as follows.

**Figure 3:**
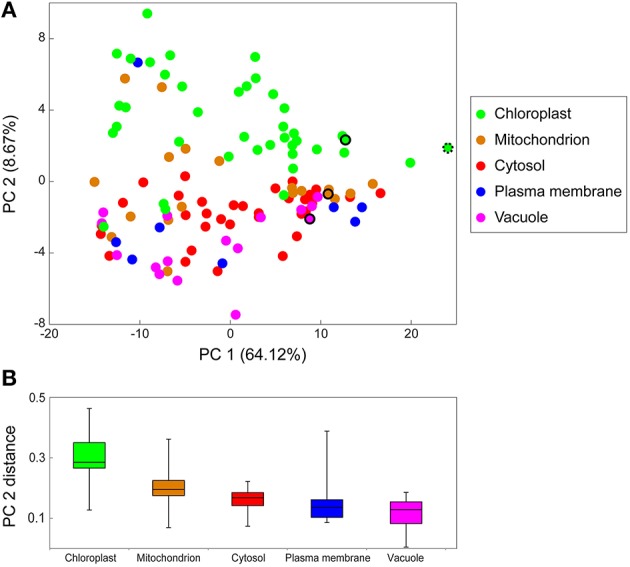
Principal Component Analysis (PCA) of marker peptide distribution in NAF. The PCA was performed using the log10 transformed intensities of the 138 marker peptides (target list Table S4) of the chloroplast (chloro), mitochondria (mito), cytosol (cyto), plasma membrane (PM), and vacuole (vacu) of the LC-MS/MS shotgun approach of the NAF-gradients. **(A)** PCA for protein distribution over the gradient. The colored circles represent one protein of the respective cellular compartment, the selected standard peptides are in the same color and encircled the organelle membrane marker peptides. MATPase, mitochondrial ATPase alpha subunit; PSI, Photosystem I iron-sulfur center; RuBisCO, Ribulose-1,5-bisphosphate carboxylase/oxygenase large subunit (both plastidial); VATPase, V (vacuolar)-type proton ATPase. **(B)** Boxplot of the PCA distance from **(A)**. PCA2 loadings of proteins of the same subcellular location were used to display the PCA distance.

Of all 15,000 identified peptides, 4,448 peptides were recognized as proteotypic peptides (only present in one protein) (Table S3). From those, a final organelle protein target list, 138 peptides that could be annotated to a specific organelle, was created (Table S4). With this, a principle component analysis was performed, visualizing the distribution profiles of the different proteins (peptides) connected to their subcellular compartments (Figure 3A). Principle component 1 (PC1) separates the peptides according to their relative abundance (intensities, normalized by the total amount of proteins analyzed). PC2 separates the proteins according to their subcellular localization (colors are related to their predicted localizations). Here, plastid proteins are clearly separated from all other subcellular compartments. However, other organelles were not as clearly separated. In some cases, proteins that have been predicted to be localized in the plasma membrane or mitochondria are more likely linked to chloroplasts as they were found in the first NAFs and clustered by the PCA. A box-whisker-plot of the PC2 distances (Figure 3B) was generated to visualize the organelle separation based on PCA loadings. The whiskers are ranging from the smallest to the largest value of the data and allow that the “subcompartmental heterogeneity” (Arrivault et al., 2014) of the different subcellular locations to be compared. The majority of chloroplast peptides separates clearly from the others, though showing a great degree of “subcompartmental heterogeneity” in form of large whiskers. Conversely, cytosolic proteins show the greatest homogeneity and the plot reveals slight differences between the mitochondrial and cytosolic distribution.

### Mitochondria Marker Peptide Evaluation

The separation power of NAF was reported to be best for plastidial, cytosolic and vacuolar compounds. Additionally, we detected a suitable marker peptide for mitochondria which we additionally tested for specificity by applying Percoll purification. Fractions 7–14 were used for further analysis as they were containing the mitochondria (Huang et al., 2014). Fractions 7–9 and 10–14 were pooled. There was a clear enrichment of mitochondrial ATPase absolute and also compared relatively to our tonoplast marker peptide of the V-ATPase (Figure S3). The absolute abundance of the M-ATPase marker peptide of the mitochondria fraction of the Percoll continuous gradient was 56 fmol, the V-ATPase marker reached a value of 7.7 fmol.

### Organelle Membrane Marker Assay (Mass Western) of Two *Pisum sativum* Cultivars

**Figure 4:**
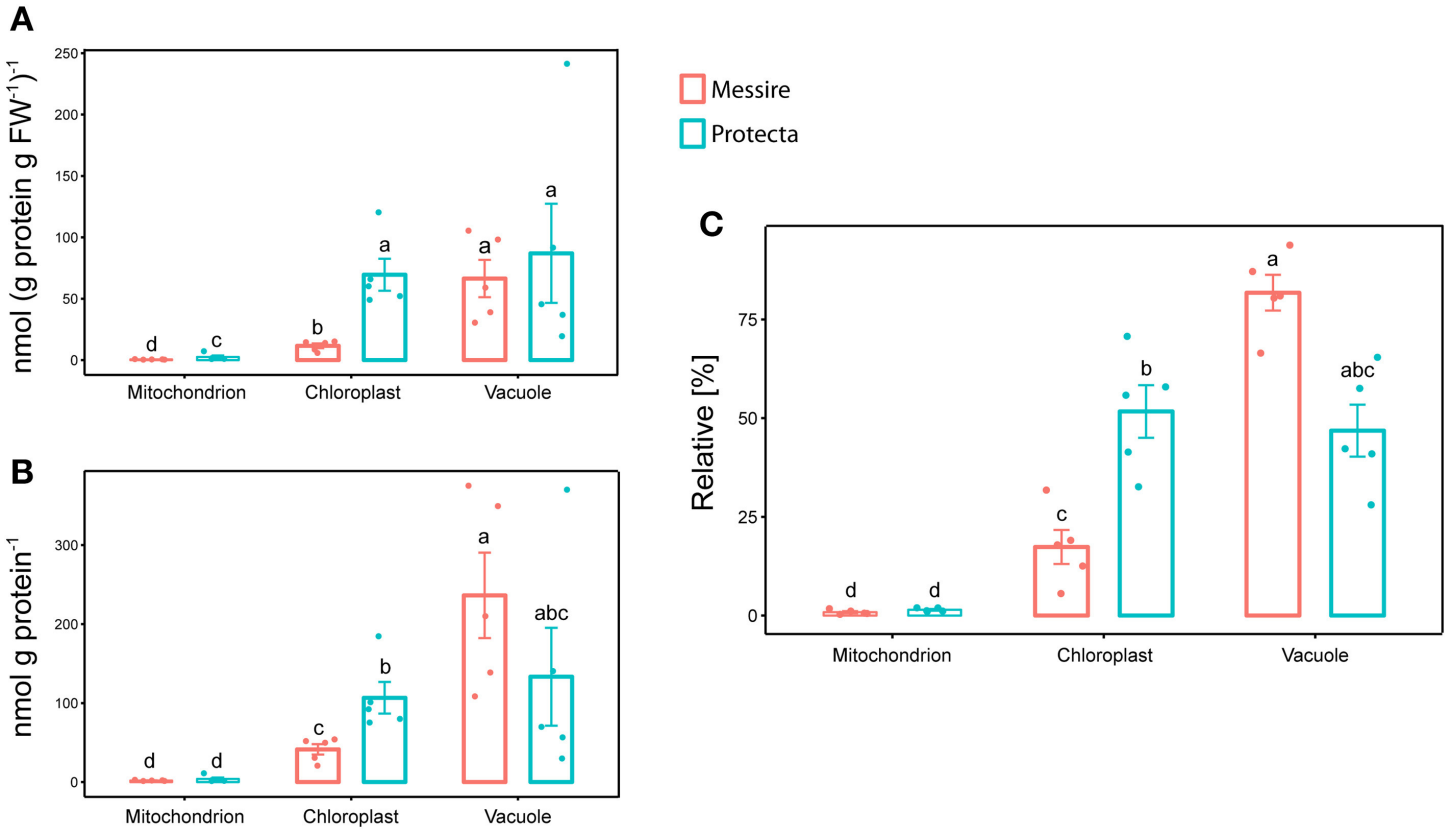
Mass Western: comparison of absolute and relative organelle-protein marker and abundances of young leaves of two pea cultivars. **(A)** Absolute protein abundances expressed as [nmol (g prot. per g FW)^−1^] and **(B)** in [nmol g prot.^−1^], **(C)** relative marker protein abundances [%]; Significant differences at *p* < 0.05 are indicated by different letters, *n* = 5 (Kruskal Wallis); error bars = standard deviation.

Initially, leaves of two *Pisum sativum* cultivars (Messire and Protecta) were used for assay development and testing. Absolute abundances of the Organelle Membrane Marker Peptides of *Pisum sativum* (OMMPOPs) were very similar, with no significant difference between the cultivars (Kuskal Wallis), when comparingthe leaf extract taken across all leaves (Figures 2D,E). The relative distributions chloroplast: vacuole: mitochondria for Protecta and Messire were about 23%: 77%: 0.5%. These marker peptide abundances showed strong similarity compared to the microscopic abundances (Figures 2B–E). In contrast, when analyzing only the youngest, fully developed leaves, the OMMPOPs revealed significant differences between the cultivars for chloroplasts, being higher for Protecta compared to Messire (Figure 4). Also the large subunit of RuBisCO was significantly higher in Protecta (Figure S4) Altogether, the OMMPOPs revealed a higher relative and absolute (nmol ^*^ g protein^−1^) abundance of chloroplasts and mitochondria in young leaves of Protecta compared to Messire and compared to all leaves, while the abundance of V-ATPase remained smaller for Protecta like it was across all leaves (Figure 4B). When considering the relation total protein per fresh weight (g/g), which was less in Messire (Figure S1), the V-ATPase abundance was comparable for both cultivars (Figure 4A). Relative abundances (sum of all measured absolute organelle abundances was set to 100%) of the OMMPOPs revealed a relative distribution of chloroplast: vacuole: mitochondria for Protecta of about 44%: 55%: 1.6% and for Messire of about 15%: 85%: 0.7% (Figure 4C). Here, the difference between the cultivars was statistically significant (*p* < 0.05) for all organelle membrane marker.

## Discussion

### Phenotyping and Quantitative Cell-Organelle Distribution by Microscopy

We compared two Pisum cultivars Protecta and Messire, as they showed several distinct phenotypic traits. Previous data described significantly higher dry mass production and green leaf area for Protecta (Turetschek et al., 2017) and also its yield seemed to be slightly enhanced (Ranjbar Sistani et al., 2017). Protecta is also known to be more resistant to the ascochyta blight pathogen (*Didymella pinodes*), compared to the susceptible cultivar Messire (Fondevilla et al., 2005; Ranjbar Sistani et al., 2017; Turetschek et al., 2017). In addition, we found internode length as well as the ratio of protein to fresh weight to be significantly (*p* < 0.05) more abundant in Protecta. The overall photosynthetic yield seemed similar between the two cultivars. However, for Messire, our physiological parameters further revealed a significantly higher efficiency of the open reaction centers during light, while its relative loss of incoming light via unregulated processes (possibly through heat formation) seemed also higher, indicative for inhibition and an overall less efficient photosynthesis (Figure S2). This was further supported by a significantly (*p* < 0.05) lower energy use efficiency during photosynthesis for Messire, which might also be reflected by higher leaf temperature differences compared to cultivar Protecta. Hence, morphological differences can at least partially be explained by physiological parameters. Linking morphological and physiological phenotypes to organelle function and distribution, such as PS efficiency to plastid abundance, is challenging as microscopy based anatomical analyses are thus far not considered in high-throughput manner. Hence, only few studies on the stoichiometry of organelle abundances in plants exist. Previously, the subcellular volumes of vacuole, chloroplasts and mitochondria were determined for barley and spinach leaves (Winter et al., 1993, 1994) as well as potato (Leidreiter et al., 1995), using light and electron microscopy. The relative stoichiometry of mitochondria to chloroplasts to vacuole ranged between 1% to 10–20% and 70–80%, respectively. Our confocal microscope (CLSM) data show a similar pattern for both Pisum cultivars, with increased values for mitochondria (~5%), chloroplasts (~25%), and the vacuole (~70%). Differences are most likely related to the species/cultivar specific variation but might partly also be due to the different techniques used. Comparing both *Pisum sativum* cultivars, the palisade parenchyma cells of all analyzed organelles of cv. Messire seemed bigger in total volume, which may be related to the higher water content, reflected by the absorbance at 940 nm. At last, the vacuoles seemed bigger in Messire. In addition, the chloroplasts volume (μm^3^) was slightly but not significantly larger, albeit not for the relative abundances. As we are dealing with living samples, a certain motion blur is inevitable during image acquisition. Thus, CLSM data gave no clear statistical differences between the cultivars, also due to the limited number of replicates. In addition, CLSM image analysis is not fully unbiased as differences in fluorescence intensity occur during imaging (bleaching) and have to be accounted for by varying the settings.

Interestingly, the 3D analyses of the cells (Figure 2 and supplemental 3D Videos 1–6) revealed an organelle partitioning pattern for several cells of Protecta. The localization of plastids and mitochondria, both, seemed to accumulate at the two distal and opposite ends of the cells. A similar phenomenon of organelle partitioning within a single cell of the palisade layer and independent of a Kranz anatomy has been described to be required for C4 Photosynthesis in *Chenopodiaceae species* (Voznesenskaya et al., 2001; Chuong, 2006). However, the phenomenon of organelle partitioning of C3 plants has thus far not been described and needs further investigation.

### Development of a High-Confidence Subcellular Peptide Marker Lists and Establishment of a High- Throughput Organelle Marker Based Mass Western Assay for *P. sativum* Leaves

Although, a body of publications exist on anatomical studies of plants, no robust method exists on the rapid and integrative stoichiometry analysis of cell organelles linking their molecular functions. Consequently, available data about specific organelle abundances, their relations and operational changes across various plant cultivars or accessions is rare in a high-throughput manner. Our approach is based on the assumption that organelle key functional and integral membrane marker enzymes, at least to some extend, reflect organelle abundance and functional readiness. A SRM based approach, using selected organelle marker proteins of *Arabidopsis thaliana*, irrespective of their localization, (such as membrane bound or intraoranellar) was previously developed(Parsons and Heazlewood, 2015; Hooper et al., 2017). The advantage of their method is the higher range of useful peptides for quantification. They claim that their method was a rough measure of relative organelle abundances, even though they did not compare it to the actual organelle abundance of their systems (cell culture vs. rosetta leaves). Nevertheless, this approach seems a good measure when comparing different systems. The Mass Western, however, combines SRM with the application of synthetic peptides in order to get absolute values for accurate protein stoichiometry calculations, allowing the comparison of different proteins to each other. Hence, we developed the organelle-membrane-marker Mass Western assay, to gain quantitative information about organelle stoichiometry related to size and function.

The non-aqueous fractionation (NAF) technique has previously been shown to be a useful tool for the distribution analysis of metabolites (Gerhardt and Heldt, 1984) and has also been applied for integrative analysis of metabolites and proteins (Arrivault et al., 2014) across organelles. By using a NAF approach, we were able to extract 138 proteotypic organelle marker peptides, suitable for further localization and SRM studies. NAF also enabled the detection of some proteinsfalsely predicted organelle association. At least, three peptides corresponding to proteins, predicted to be associated with the plasma membrane and mitochondria, have to be reassigned to have chloroplast association (Table S4). Essentially, we used NAF for a specific selection of organelle-membrane marker peptides of *P. sativum* (OMMPOPs) leaves. Membrane proteins can be best related to organelle surface abundances. Hence, we considered the abundance of these proteins to be in good proportion to size and function of their organelles. The selection of our marker peptides according to their robust mass spectrometric detection properties and proteotypicality of membrane integral proteins, restricted the number of suitable peptides to a minimum, and was especially challenging for the vacuole (Parsons and Heazlewood, 2015; Hooper et al., 2017). Thus, stringent selection resulted in one membrane-marker peptide per organelle and the possibility to quantify three different organelles in one single LC-MS/MS analysis without the necessity for any additional sample enrichment or dilution steps and without the need for SRM. We believe that in this particular case one peptide per organelle is enough for screening as they were carefully selected, well representative and robust for the detection of stoichiometric relevant organelle differences/changes.

In general, V-ATPase are not always restricted to vacuoles. Specific isoforms were also found in the plasma membrane and the trans-Golgi network (Nishi and Forgac, 2002; Dettmer et al., 2006). Even though, it cannot fully be exclude that this isoform may also be found unspecific in other membranes, it is rather unlikely as the transport of these functionally important membrane proteins to their target membrane need to be controlled. Furthermore, we found our V-ATPase marker-protein with one proteotypic peptide only in the vacuolar fraction of the NAF gradienta nd thus are confident of the specificity of this isoform. For mitochondria, the discrimination power of NAF is limited (distributed across several fractions). Thus, we decided to check the specificity of the mitochondrial marker peptide against a purified mitochondria preparation (Huang et al., 2014). The result proved the high quality of this marker peptide as the abundance was strongly increased in the mitochondrial fraction and also compared to the V-ATPase marker peptide, which we used as a reference. Altogether, our results OMMPOPs-Mass Western comprises of proteotypic and organelle-membrane specific peptides, with robust and sensitive properties for MS.

### Evaluation of Quantitative Data From Microscopy and Mass Western

In order to test the OMMPOPs-Mass Western for their potential to reflect organelle abundances and reveal differences between phenotypes, we applied the assay to crude extracts of two *P. sativum* cultivars, varying in growth performance and photosynthetic efficiency. When comparing the results of the relative organelle distributions from literature with our CLSM and Mass Western data, a good fit with highest abundance values for vacuole, chloroplasts and mitochondria was observed. The abundance of the vacuole membrane protein marker was also more prominent in cv. Messire in accordance with the microscopic data of slightly higher volumes. Interestingly, when testing the young leaves only, this observation became evident; Protecta vacuole marker abundance was significantly (*p* < 0.05) lower compared to Messire. In contrast, chloroplast and mitochondria marker were distinctively (*p* < 0.05) more abundant in cv. Protecta. The results suggest that the larger growth performance and higher protein to leaf fresh weight values of cv. Protecta are related to higher abundances of the marker-proteins of the two important energy processing organelles of the young leaves, where protein abundance is often a good proxy for their activity (Lehmann et al., 2008). A better photosynthetic efficiency, especially of the young leaves of cv. Protecta, is further supported by the significantly higher abundance of the large subunit of RuBisCO. The data imply that in those leavesplastids and mitochondria are more abundant in Protecta compared to Messire. The relative organelle marker peptide abundances of young leaves of Protecta are striking, as the chloroplasts are relatively similar abundant than the vacuole. Here, the enhanced abundance of the marker peptide seems to be linked to a higher functional rather than size related proportion. Interestingly, when calculating the specific protein abundance (nmol per g protein; not taking the fresh weight into account) the vacuole marker abundance of cultivar Messire increase compared to that of Protecta, however, not as significantly as reflected by the relative abundance. This finding supports the interpretation of the physiological data, indicating a higher water content in the leaf tissue of cultivar Messire. Taken together, the OMMPOPs-Mass Western seemed effective in cultivar difference analysis and opened a couple of new questions. However, OMMPOPs seemed to reflect relative organelle abundances at least to a certain extent. The approach is effective in separating cultivars according to functional organelle properties that can be explained by their morphological and physiological differences.

### Pros and Cons of the Mass Western Assay

Membrane marker enzyme activity assays are traditionally used for the determination of purity after organelle enrichment studies (Yoshida et al., 1983). Hence, the OMMPoPs-assay also serves as a sensitive organelle enrichment analysis. The OMMPoPs-Mass Western is not *per se* limited to the three major organelles (vacuole, chloroplasts, and mitochondria) and target peptides of other organelle markers may work as well. However, major challenges for this are for instance the limited subcellular localization studies of the *P. sativum* proteome, the limited NAF separation power for other organelles and their low abundances, as well as the difficulty of cross-evaluation with microscopy since organelle specific visualization is also limited. We have chosen organelle marker peptides according to their properties for mass spectrometry, which resulted in the selection of high abundance membrane enzymes. The advantage of this is that it can be run as simple LC-MS/MS analysis without needing SRM. Using SRM is particularly important when quantifying low abundance proteins (Wienkoop and Weckwerth, 2006). The advantage of the OMMPoPs is that the target peptide signals are very robust and to be expected among the most abundant signals of a Pisum leaf crude extract. However, abundance of those membrane marker proteins might change upon environmental perturbations, or during development, which can be related to either a change in its abundance without or due to changes in organelle abundance or because of both. It seems evident that membrane-protein and organelle abundance changes occur in parallel, especially when comparing organelles of the same cultivar and tissue in response to environmental perturbations or leaves of varying developmental stages. Hence, it should be clear that a change in marker peptide abundance will indicate an adjustment of organelle function which although not necessarily directly linked, can serve as a good indicator of changes in organelle abundance. In fact, the choice of marker proteins that are not responsive to regulation has proven itself to be difficult (Parsons and Heazlewood, 2015). Nevertheless, they recommended to avoid light harvesting complex proteins, as these are highly responsive to their environment. However, especially the challenging search for chloroplast membrane marker that have no regulatory function during environmental changes, opens the question, if this is actually required. Nevertheless, in order to better evaluate between functional and/or organelle-abundance related protein-abundance changes, a set of different protein markers is necessary. Since the choice of membrane proteins and peptides is highly limited, the focus should be directed toward structural membrane proteins, which possibly are not involved in plant adaptation processes. For instance, mitofilin as structural constituent of the inner mitochondrial membrane (John et al., 2005; Kolli et al., 2018) presents itself as an interesting target for the characterization of actual mitochondria organelle abundance changes. In fact, we identified a protein (generic|frv2_81024|Pisum_sativum_v2_Contig4490_2) with a high similarity (83%) to a MICOS (mitochondrial contact site and cristae organizing system) protein complex subunit (MIC60; XP_024629043.1) of *Medicago truncatula*. However, the protein was by a factor of 30 less abundant and thus also less robust for mass spectrometric analysis compared to our chosen target. In this case, a simple 1D shotgun analysis of a crude extract would not allow for the quantification of all three organelles due to a restricted order of magnitude for MS (Wienkoop and Weckwerth, 2006; Lyon et al., 2014). Besides these critical aspects that have to be considered when using protein marker for organelle abundance estimation, there are clear advantages of using the OMMPoPs approach. The results of the relative and absolute organelle membrane marker abundances analysis revealed significant differences between the two cultivars and between developmental stages of the leaves, which is the major aim of high-throughput phenotyping. It is noteworthy that the mitochondrial marker peptide, selected here, also matches other model legumes such as *Medicago truncatula* (tr|A0A072TFS9), *Lotus japonicus* (YP_005090487), and *Glycine max* (YP_007516887). In addition, the chloroplast photosystem I (PSI) marker matches to *Lotus japonicus* (NP_084846) and *Glycine max* (YP_538817) PSI protein. Thus, the Mass Western can be applied at least partly to other legumes and probably also to several other plant species. The presented OMMPOPs Mass Western is robust and optimized for high-throughput and suitable for estimating differences in organelle protein stoichiometry of *Pisum sativum* cultivars and/or upon environmental perturbations within the same cultivar(s).

## Conclusion

Taken together, NAF gradients were successfully applied to separate proteins belonging to the subcellular compartments chloroplasts, mitochondria and vacuole and turned out to be suitable for (re)annotation of plastidial proteins that have wrongly been annotated to other subcellular localizations before. NAFs can therefore serve as a tool to find proteotypic marker peptides for targeted protein quantification approaches such as the Mass Western. We further conclude that CLSM is suitable for organelle visualization however not appropriate for high throughput quantification and large scale screening. In addition, a high replicate number would be required for statistical relevance. However, microscopy is inevitable, when it comes to actual organelle stoichiometry, number or size changes, and cell partitioning pattern recognition. In contrast, the Mass Western can be used in a high-throughput manner. In addition, a better discrimination between cultivars can be achieved with a relatively low number of biological replicates. Overall the organelle marker abundances provide information on the functional and on the relative organelle abundance level that seems very suitable for the differentiation between cultivars and within the same cultivar in response to environmental perturbations. It supports explanations of phenotyp/genotype relations in a high-throughput manner. This study also highlights the importance of collecting data from several approaches (morphology, anatomy, physiology, and proteomics) for comprehensive phenotyping and data interpretation.

## Author Contributions

SS performed experiments and protein data analysis and was involved in manuscript writing. DH performed microscopic image data analysis. GB performed the physiological and morphological phenotyping. TN supported the NAF analysis. IL was supporting and supervising the microscopy and was involved in manuscript writing. SW conceived the study, was in charge of overall direction and planning, performed data analysis and wrote the manuscript. All authors provided critical feedback and helped shape the research, read and approved the manuscript.

### Conflict of Interest Statement

The authors declare that the research was conducted in the absence of any commercial or financial relationships that could be construed as a potential conflict of interest.

## Supplementary Material

Figure S1 Morphological phenotypes of the *Pisum sativum* cultivars Protecta (left) and Messire (right). Length of internodes and leaf weight *n* = 5 biol. replicates, error bars = standard error, *p* < 0.05 (Kruskal Wallis). ^**^*p* < 0.01, ^***^*p* < 0.005.

Figure S2 Physiological phenotyping of the *Pisum sativum* cultivars Protecta and Messire. **(A)** Chlorophyll content, measured with a Minolta SPAD, **(B)** chlorophyll fluorescence (Fv/Fm), **(C)** non-photochemical quenching (NPQt), **(D)** energy loss to thermal dissipation (PhiNO), **(E)** leaf water content (absorbance at 940 nm) and **(F)** leaf surface cooling by evapotranspiration (leave temperature difference). *n* = 30, error bars = standard error, confidence intervals at 95% Kruskal-Wallis ^*^*p* < 0.05, ^***^*p* < 0.005.

Figure S3 Mitochondrial marker peptide quality and specificity test using a Percoll gradient for mitochondria enrichment. Mitochondria were enriched according to Huang et al. (2014) and the marker peptide abundances [fmol μg of protein^−1^] of the two ATPases analyzed. Fractions 7–9 and 10–14 were pooled. The latter was the region of mitochondria enrichment. *F* = pooled Percoll fraction.

Figure S4 Time dependent absolute changes of RuBisCO abundances of young leaves of pea cultivars. Absolute abundance [nmol g protein^−1^] and [nmol (g protein g FW^−1^) ^−1^] of cultivars Protecta and Messire. Asterisk if difference of abundance statistically significant, ^*^*p* < 0.05 (Kruskal-Wallis), *n* = 5; error bars = standard error, (FW = fresh weight).

Table S1 **(A)** Selected target peptide list for the Mass Western; **(B)** Heavy isotope labeled standard peptides. In brackets those amino acids that were labeled heavy isotopic.

Table S2 Avarage abundance values of the confocal microscopy analyses for volumes and areas of the analyzed organelles of *Pisum sativum* leaf cells for cultivar Protecta and Messire.

Table S3 Information of selected proteotypic peptides from all identified *Pisum sativum* leaf proteins and their corresponding peak intensities (LFQs, MaxQuant) in 9 fractions each of the 10 different non-aqueous fractionations (NAF).

Table S4 List of 138 proteotypic target peptides (proteins) with subcellular localization analysis.

Videos 1–6 The videos are an additional information to Figure 2 and show the confocal microscope 3D reconstructions and surface rendering of the palisade cells of *P. sativa* cultivars of Messire and Protecta in more detail. Labeling as also described in the methods: cytoplasm labeled with fluorescein, mitochondria labeled with MitoTracker orange^®^, and autofluorescence of chlorophyll. The vaculole cavity was labeled in yellow. Videos Messire 1–3 represent cells 1 to 3 and Protecta 1–3 cells 1 to 3 of the two cultivars, respectively.
