# Supplementary material for: Subcellular Phenotyping: Using Proteomics to Quantitatively Link Subcellular Leaf Protein and Organelle Distribution Analyses of Pisum sativum Cultivars
Source: Front Plant Sci. 2019 May 17;10:638. doi: 10.3389/fpls.2019.00638 (PMC6534152; doi:10.3389/fpls.2019.00638)
Supplement: Figure S1 — Morphological phenotypes of the Pisum sativum cultivars Protecta (left) and Messire (right). Length of internodes and leaf weight n = 5 biol. replicates, error bars = standard error, p < 0.05 (Kruskal Wallis). **p < 0.01, ***p < 0.005. [file Data_Sheet_1.docx]

Supplementary Material

Subcellular Phenotyping: Using proteomics to quantitatively link subcellular leaf protein and organelle distribution analyses of *Pisum sativum* cultivars

Sebastian Schneider, Dominik Harant, Gert Bachmann, Thomas Nägele, Ingeborg Lang and Stefanie Wienkoop^1*^

*** Correspondence:** Dr. Stefanie Wienkoop: stefanie.wienkoop@univie.ac.at

**
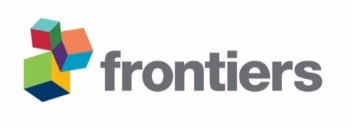
**

**
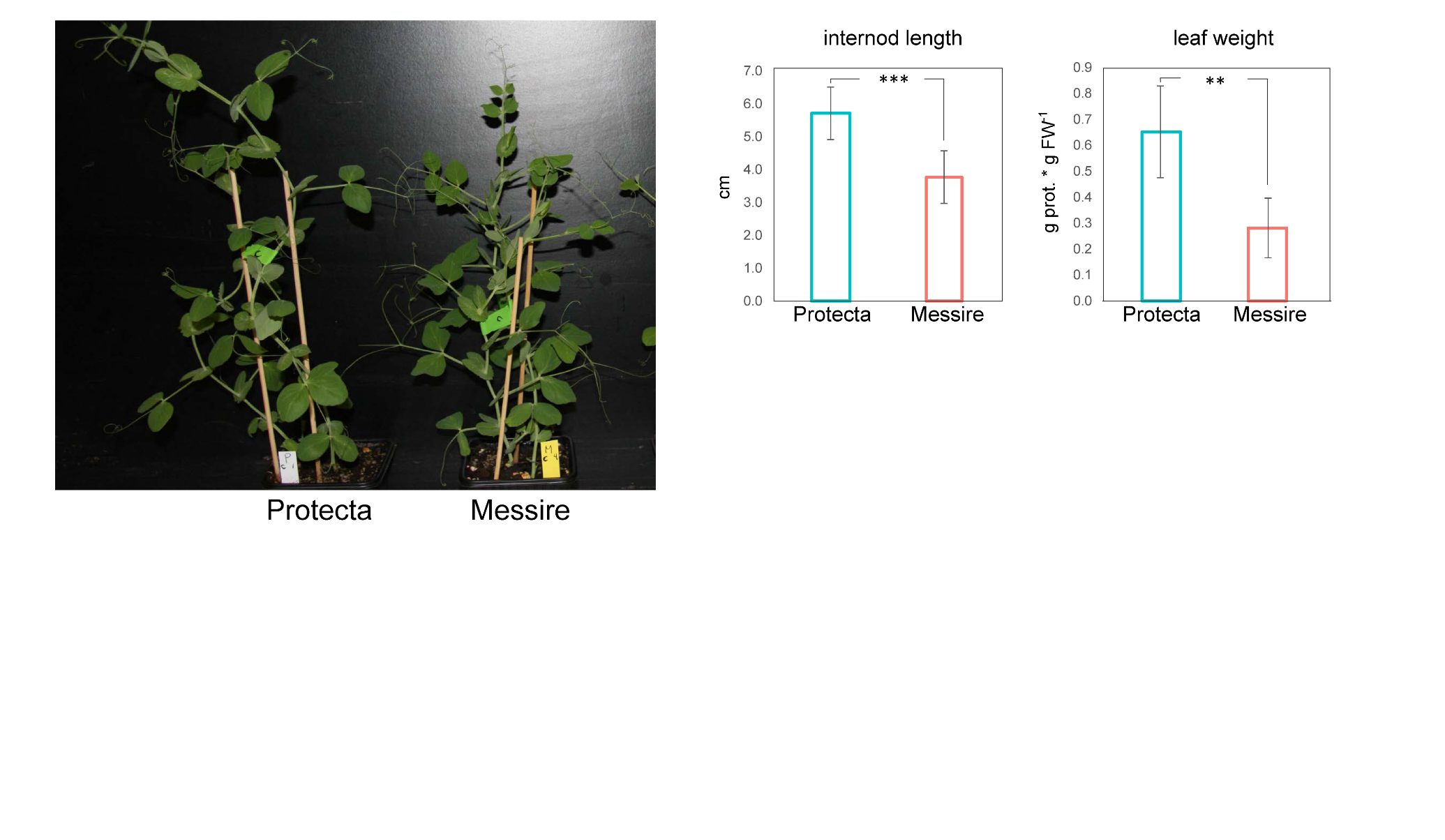
**

Figure S1 | Morphological phenotypes of the Pisum sativum cultivars Protecta (left) and Messire (right). Length of internodes and leaf weight n = 5 biol. replicates, error bars = standard error, ** p < 0.01, *** p<0.001 (Kruskal Wallis).

**
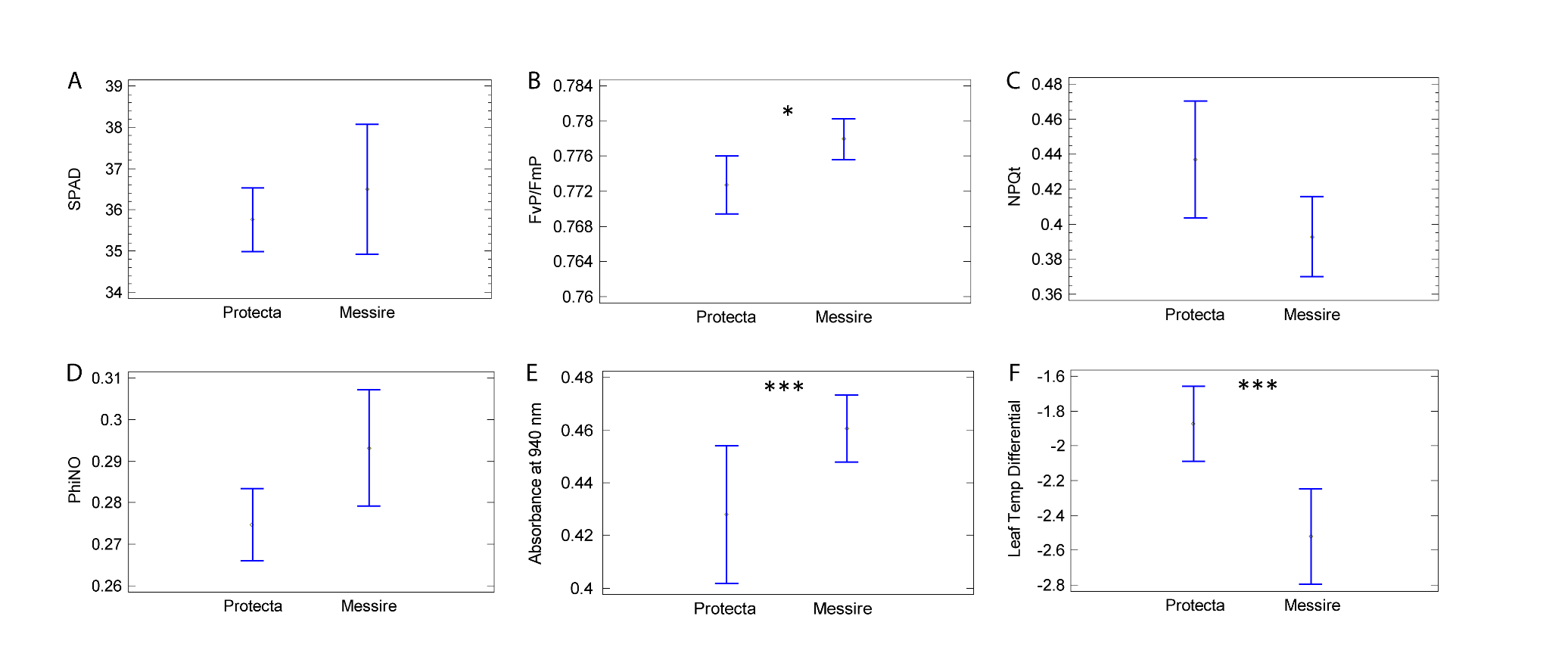
**

Figure S2 | Physiological phenotyping of the Pisum sativum cultivars Protecta and Messire. (A) Chlorophyll content, measured with a Minolta SPAD, (B) chlorophyll fluorescence (Fv/Fm), (C) non-photochemical quenching (NPQt), (D) energy loss to thermal dissipation (PhiNO), (E) leaf water content (absorbance at 940 nm) and (E) leaf surface cooling by evapotranspiration (leave temperature difference). ). n= 30, error bars = standard error, confidence intervals at 95% Kruskal-Wallis * = p<0.05, *** = p<0.005.

**
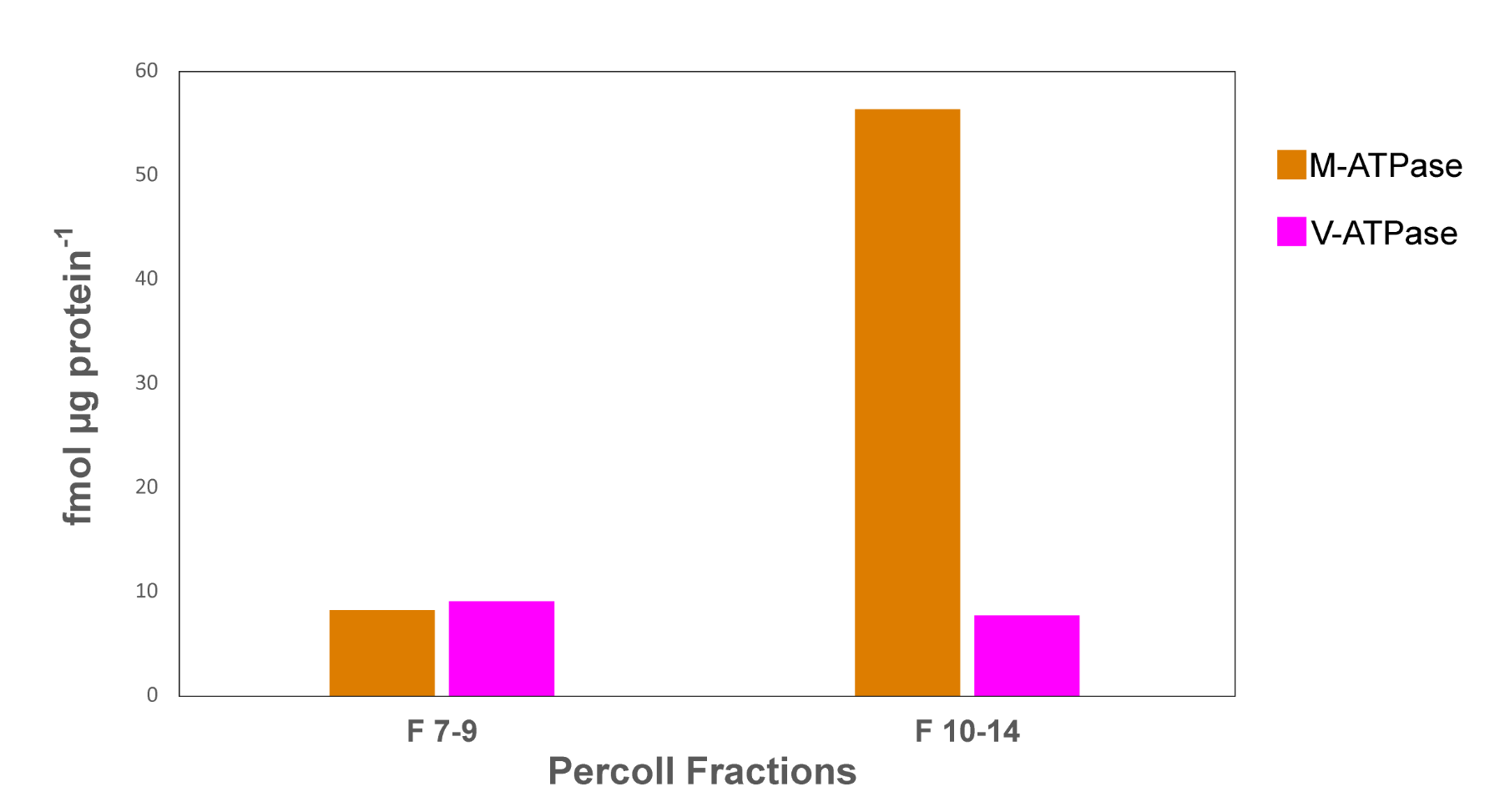
**

Figure S3| Mitochondrial marker peptide quality and specificity test using a Percoll gradient for mitochondria enrichment. Mitochondria were enriched according to Huang *et al.* (2014) and the marker peptide abundances [fmol µg of protein^-1^] of the two ATPases analysed. Fractions 7-9 and 10-14 were pooled. The latter was the region of mitochondria enrichment. F = pooled Percoll fraction.


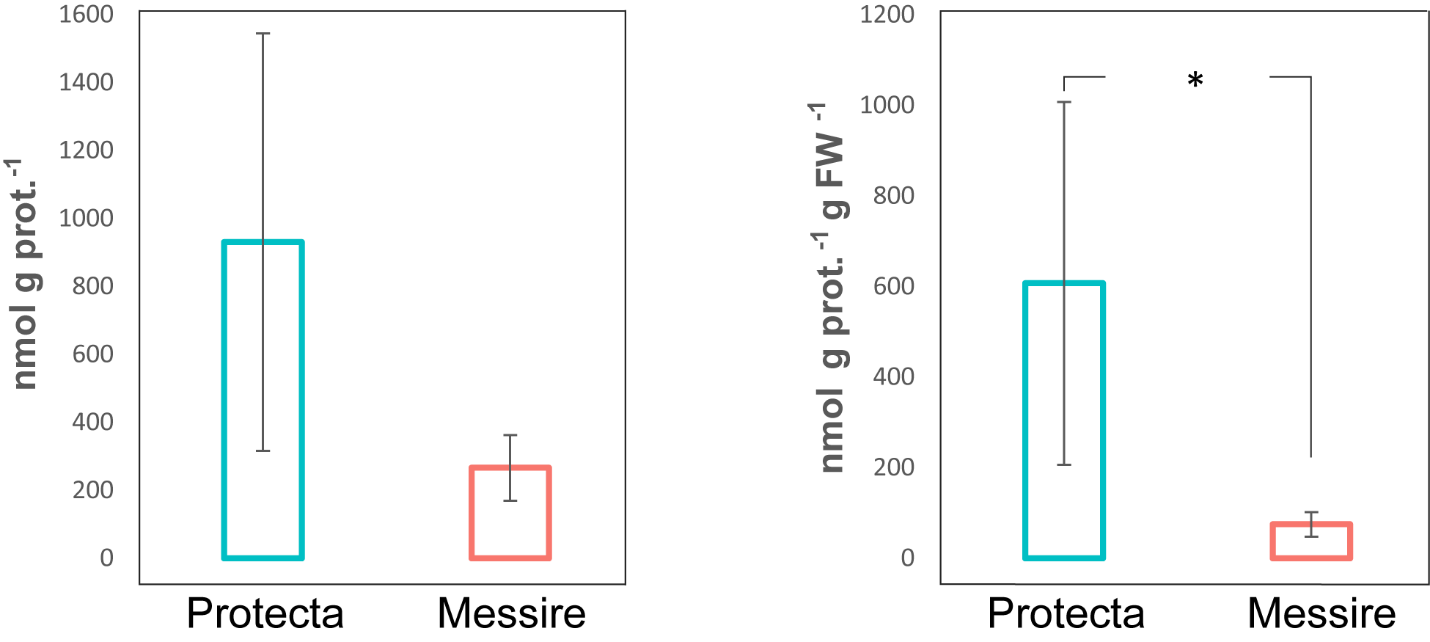


Figure S4 |Time dependent absolute changes of RuBisCO abundances of young leaves of pea cultivars. Absolute abundance [nmol g protein^-1^] and [nmol (g protein g FW^-1^) ^-1^] of cultivars Protecta and Messire. Asterisk if difference of abundance statistically significant, *= p<0.05 (Kruskal-Wallis), n=5; error bars = standard error, (FW = fresh weight).

| **Table S1 A. Selected target peptide list for the Mass Western** | | | |  |
| --- | --- | --- | --- | --- |
| Protein description | Identifier | Peptide sequence | Organelle | Subcellular localization |
| RuBisCO (LS) | 3893097 | EIKFEFPAMDTL | chloroplast | stroma |
| Photosystem I iron-sulfur center | 295136986 | VYLWHETTR | chloroplast | tylakoid membrane |
| V-type proton ATPase | frv2_80154 | ISEDVVAMLLK | vacuole | tonoplast |
| ATPase alpha subunit | 543866 | AVDSLVPIGR | mitochondrion | inner membrane |
| **Table S1 B. Heavy isotope labeled standard peptides. in brackets those amino acids that were labelled heavy isotopic.** | | | | |
| Name | Peptide Sequence | Peptide mass (light) | Peptide mass (heavy) | |
| RuBisCo | EIKFEFPAMDT(L) | 720.857603 | 724.366185 |  |
| PSI Iron Sulfur Centre | VYLWHETT(R) | 602.809105 | 607.813239 |  |
| V_ATPase | ISEDVVAMLL(K) | 609.344131 | 613.351231 |  |
| M_ATPase | AVDSLVPIG(R) | 513.800748 | 518.804883 |  |
| EP_A | LVNE(L)TEF(A)K |  | 587.831102 |  |
| EP_B | LVNELTEF(A)(K) |  | 588.32962 |  |
| QP | LVNELTEF(A)K |  | 584.32 |  |
| **concatenated:** |  |  |  |  |
| EP_A+ M_ATPase + RuBisCO | LVNE(L)TEF(A)KAVDSLVPIG(R)EIKFEFPAMDT(L) | |  |  |
| EP_B + PSI + V_ATPase | LVNELTEF(A)(K)VYLWHETT(R)ISEDVVAMLL(K) | |  |  |
